# Preliminary Findings of Needs of Older Cambodians in Three Provinces

**DOI:** 10.1093/geroni/igaa057.999

**Published:** 2020-12-16

**Authors:** Kakada Kuy

**Affiliations:** Washington University St Louis, University City, Missouri, United States

## Abstract

The most recent national survey of older Cambodians was conducted in 2004. To further our understanding of Cambodia’s older adults, new data are required. This primary data collection study aimed to assess physical, psychological and social needs of older Cambodians and examine gender differences in those needs. We collected data in 2019 in three provinces in the northwestern region of Cambodia (N=210). Due to older Cambodians’ higher level of illiteracy, research assistants filled out paper surveys for them during individual interviews. Then responses were transferred into an e-survey database for data management. Physical needs were examined with self-rated health and health complaints. Psychological wellbeing and depression scores were used to measure psychological needs. For social needs, we examined social support using Social Network and Social Support scale. More than half of older Cambodians had less than good physical health and reported numerous health symptoms. Yet, three quarter reported higher than average psychological wellbeing and a low level of depression. Some of the samples showed modest level of social support in their family and community. Furthermore, independent two-sample T-tests showed that older men and women did not differ in physical and social needs (p<.001). Older women, however, did poorly in psychological needs compared to men (p<.001). Men also had lower level of depression and better psychological wellbeing (p<.001). Future studies shall further investigate other aspects of Cambodia’s aging population in which gender differentials may also exist.

